# Intramyocardial left anterior descending unroofing using a minimally invasive off-pump approach

**DOI:** 10.1007/s12471-024-01866-8

**Published:** 2024-04-05

**Authors:** Mara-Louise Wester, Annemiek M. J. De Vos, Peter Elsman, Joost Ter Woorst, Ferdi Akca

**Affiliations:** 1 Department of Cardiothoracic Surgery, Catharina Hospital, Eindhoven, The Netherlands; 2 Department of Interventional Cardiology, Catharina Hospital, Eindhoven, The Netherlands; 3 Department of Interventional Cardiology, Jeroen Bosch Hospital, Den Bosch, The Netherlands

A 50-year-old male had been suffering from angina pectoris functional class III/IV for the past 9 years. Initially, medical therapy was attempted, but the patient continued to experience disabling angina. The coronary angiogram revealed an intramyocardial left anterior descending artery (LAD) extending from the second diagonal branch to the apex (Fig. 1a). Echocardiography indicated normal ventricular function. The patient was accepted for minimally invasive unroofing surgery. During the procedure, a left-sided mini-thoracotomy was performed and the LAD course was identified. The entire intramyocardial course could be unroofed using minimally invasive off-pump techniques (Fig. 1b, and see Video 1 in Electronic Supplementary Material). The LAD diameter was significantly larger than initially suspected. When we compared the pre- and postoperative coronary angiograms, we noticed the LAD diameter had increased 2–3 times (Fig. 1). The postoperative course was uneventful and the patient was discharged after 3 days. Follow-up assessments revealed complete remission of chest pain. Minimally invasive unroofing surgery can be a solution for selected patients with this complex disease [1].

**Fig. 1:**
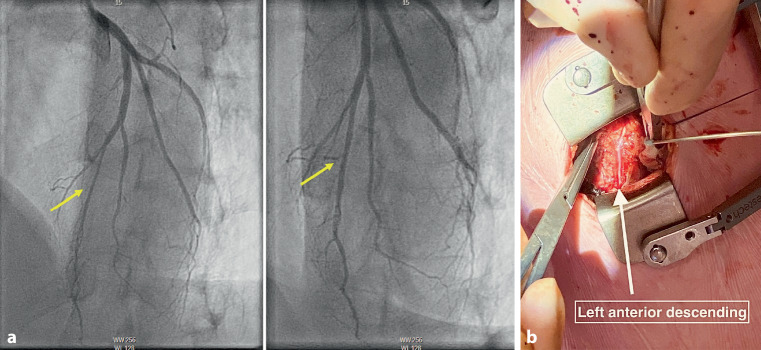
**a** Preoperative (left panel) and postoperative (right panel) coronary angiograms. Yellow arrows indicate left anterior descending artery (*LAD*). Postoperatively, the LAD diameter is evidently larger. **b** (Minimally invasive) surgical view of LAD after coronary unroofing. The entire course of the LAD is now clearly visible

## Supplementary Information

**Video 1** Operative video showing entire course of left anterior descending artery after unroofing
